# The Role of Mitochondrial Reactive Oxygen Species in Cardiovascular Injury and Protective Strategies

**DOI:** 10.1155/2016/8254942

**Published:** 2016-04-21

**Authors:** Danina M. Muntean, Adrian Sturza, Maria D. Dănilă, Claudia Borza, Oana M. Duicu, Cristian Mornoș

**Affiliations:** ^1^Department of Pathophysiology, Victor Babeș University of Medicine and Pharmacy, 2 Eftimie Murgu Square, 300041 Timișoara, Romania; ^2^Center for Translational Research and Systems Medicine, Victor Babeș University of Medicine and Pharmacy, 2 Eftimie Murgu Square, 300041 Timișoara, Romania; ^3^Department of Cardiology-2nd Cardiology Clinic, Victor Babeș University of Medicine and Pharmacy, 2 Eftimie Murgu Square, 300041 Timișoara, Romania

## Abstract

Ischaemia/reperfusion (I/R) injury of the heart represents a major health burden mainly associated with acute coronary syndromes. While timely coronary reperfusion has become the established routine therapy in patients with ST-elevation myocardial infarction, the restoration of blood flow into the previously ischaemic area is always accompanied by myocardial injury. The central mechanism involved in this phenomenon is represented by the excessive generation of reactive oxygen species (ROS). Besides their harmful role when highly generated during early reperfusion, minimal ROS formation during ischaemia and/or at reperfusion is critical for the redox signaling of cardioprotection. In the past decades, mitochondria have emerged as the major source of ROS as well as a critical target for cardioprotective strategies at reperfusion. Mitochondria dysfunction associated with I/R myocardial injury is further described and ultimately analyzed with respect to its role as source of both deleterious and beneficial ROS. Furthermore, the contribution of ROS in the highly investigated field of conditioning strategies is analyzed. In the end, the vascular sources of mitochondria-derived ROS are briefly reviewed.

## 1. Introduction

Ischaemia/reperfusion (I/R) injury of the heart represents a major health burden mainly associated with acute coronary syndromes. Each year, myocardial infarction (MI) is responsible for the death of millions of persons and, more importantly, due to the aging of the population, represents the first cause of chronic heart failure worldwide [1]. Thus, it is not surprising that it has been predicted, already 10 years ago, that more than 40% of US population will suffer from heart failure as end stage of cardiovascular pathologies by the year 2030 [2].

Timely coronary reperfusion by either thrombolysis or primary coronary artery angioplasty has become the established routine therapy in patients with ST-elevation MI (STEMI) which effectively decreases infarct size and reduces mortality [3]. Paradoxically, restoration of the blood flow into an ischaemic area is always accompanied by myocardial injury [4]. In fact, several distinctive pathophysiological changes have been systematically associated with revascularization. These changes, collectively denominated “reperfusion injury,” comprise both (i)* reversible (sublethal)* events such as* reperfusion-induced arrhythmias* and* myocardial stunning* (prolonged but fully reversible contractile dysfunction) and (ii)* irreversible (lethal)* ones, namely, the* accelerated necrosis* in tissue that has been already irreversibly injured (the “oxygen paradox”) [5], the induction of* microvascular obstruction* (responsible for the no-reflow phenomenon), and the* lethal reperfusion injury* (death of cardiomyocytes that were potentially viable at the end of the ischaemic event, that is, prior to reperfusion) (reviewed in [6–8]). Although the existence of this last major event was at the time a matter of debate [9, 10], substantial experimental evidence supported, firstly, the fact that irreversible reperfusion injury (through necrosis, apoptosis, and autophagy) exists [11] and, secondly, the concept that early reperfusion represents a window of opportunity for the delivery of adjunctive therapies capable of preventing cardiomyocyte death [12–14].

During the past four decades, a tremendous research effort was put forward to elucidate the pathophysiology of I/R injury and identify strategies that are able to provide cardioprotection at reperfusion, that is, to enhance the amount of myocardium salvaged by timely restoration of the blood flow [6, 7, 14, 15]. In this respect, the mitochondrion is the organelle that has been unanimously indicated as the major culprit responsible for the development of cardiomyocyte death [11, 16–19] and, also, the primary target in protecting the heart against the deleterious effects of reperfusion injury [20–23].

Among the main mechanisms that underlie mitochondrial dysfunction and, ultimately, cardiomyocyte death in the setting of I/R injury, namely, calcium dysregulation, ATP depletion, release of proapoptotic proteins, and oxidative stress, the last issue, that is, excessive formation of reactive oxygen species (ROS) with the subsequent damage of cell constituents, plays a central role as it is able to trigger and/or potentiate each of the other mentioned mechanisms [24].

However, in neither field has the well-known principle stated by Paracelsus “*dosis sola venenum facit*” (“*the dose alone makes the poison*”) been more true as in the case of redox biology. Indeed, while increased oxygen radical production is the central mechanism involved in postischaemic myocardial injury, minimal ROS formation is critical for the redox signaling of cardioprotection (reviewed in [25–31]).

Basic cardiovascular research has witnessed the discovery of a myriad of ways to protect the heart/cardiomyocytes in various experimental models of I/R injury. Despite the fact that clinical application of these strategies has been thus far limited [32], the development of specific molecules targeting mitochondria of living cells for therapeutic gain is a rapidly evolving field and a number of drugs have already entered clinical testing [21, 33–36].

Mitochondria dysfunction associated with postischaemic myocardial injury is further described and ultimately analyzed with respect to its role as source of both deleterious and beneficial reactive oxygen species in the setting of I/R injury and for cardioprotective signaling, respectively. Last but not least, the potential vascular sources of mitochondria-derived ROS are briefly reviewed.

## 2. Mitochondria Dysfunction in Ischaemia/Reperfusion Injury: Historical Perspective

Mitochondria occupy a fixed fractional volume (~21% of the total heart mass) in mammalians and are strategically placed in the vicinity of myofibrils to ensure the delivery of a huge amount of ATP (ten times the cardiac mass) which is largely generated via oxidative phosphorylation and required for the myocardial contraction that occurs within a wide workload range [37]. Since the heart is strictly dependent on aerobic metabolism, it is not surprising that cardiac pathology is intimately intricated with mitochondrial impairment in the setting of myocardial I/R injury. Moreover, heart is primarily a postmitotic organ and, therefore, the death of cardiomyocytes is the major phenomenon that underlies this organ pathology.

Most of our research knowledge regarding the structural and biochemical changes elicited by experimental ischaemia and reperfusion comes from the pioneering studies started in the late 60s by Robert Jennings. In his seminal papers, he provided a clear definition of* lethal ischaemic injury* as being “the ischaemic injury of sufficient severity and duration that the involved cells will continue to degenerate and become necrotic despite reoxygenation by reperfusion of arterial blood” [38, 39]. This definition points to the* gradual* pattern of the process, in which irreversibly injured ischaemic cardiomyocytes will ultimately progress to the loss of membrane integrity and necrotic cell death. At the end of prolonged ischaemic episodes (e.g., 40 min in dogs), mitochondria in irreversibly injured cells contain one or more small (80–150 *μ*m) amorphous matrix densities, being ascribed as “the most reliable indicator of irreversibility” [40, 41]. Interestingly, these dense matrix deposits (consisting primarily of lipids and little calcium) were considered a characteristic feature not only of ischaemia-related irreversible injury of the heart but also of drug and toxic-induced injury in liver and kidney [41]. Besides the gradual pattern of progression to death, when reperfused* in vivo* after prolonged periods of ischaemia, cardiomyocytes undergo an* abrupt* irreversible injury characterized by hypercontracture and a* rapid* increase in permeability of the sarcolemmal membrane responsible for the release of intracellular enzymes [42], observations relevant for the phenomenon of* lethal reperfusion injury*. Mitochondria in these cells showed diffuse swelling and accumulated a second type of matrix densities, distinct from the amorphous ones already present at the time of reperfusion; these granular dense bodies contained a large amount of calcium precipitated as an initially undefined form of calcium phosphate [40, 41]. As noticed in a critical review, the most important finding of the classical studies was that the hallmark events of irreversible injury, that is, hypercontracture and calcium overload,* required functional (coupled) mitochondria* in order to occur [43]. Indeed, hypercontracture of sarcomeres into contraction bands [44] and mitochondrial accumulation of calcium as well as the enzymes release were all lessened by inhibiting mitochondrial respiration with the subsequent decrease in ATP synthesis (reviewed in [45, 46]). Accordingly, it was evident already almost 4 decades ago that restoration of mitochondrial function in myocardial cells after severe ischaemia was the major culprit for the double-edged sword effect of reperfusion, since ATP production via oxidative phosphorylation was mandatory for the recovery of cardiomyocytes but appeared to also contribute to the postischaemic cell death. Despite the thoroughly performed experiments, it should be mentioned that Jennings and Ganote refrained themselves from affirming a causal relationship between the “observed changes in mitochondrial structure and function and the death of the myocardial cell” due to the technical limitations at that time [40].

Similarly, these authors acknowledged the role of excessive production of oxygen free radicals at reperfusion and their toxic effects on both myocardium and vasculature; however, they admitted only the possibility that in the case of irreversibly injured myocytes “free radicals might accelerate the degradation of dead cells, but not kill any cells which were otherwise viable” [42]. Moreover, 15 years ago, both the existence and, more importantly, the clinical relevance of the reperfusion injury were strongly questioned [47].

The disrupted mitochondrial electron system has been already identified by the mid-70s as a potential source of oxyradicals (in particular superoxide) in the setting of I/R injury; the process is further contributed by a decrease in the free radical scavenging capacity due to the loss of mitochondrial reduced superoxide dismutase and reduced glutathione (reviewed in [48]). The uncontrolled reactivation of mitochondria upon oxygenation with subsequent ROS generation has also been considered responsible for the peroxidation of cardiac lipids, increased sarcolemmal permeability, and enzyme release; the events have been prevented in the presence of either superoxide dismutase or reduced glutathione administered at the end of hypoxia and during reoxygenation [49].

Indeed, as Halliwell mentioned, it was the time when “*the field of free radicals and antioxidants was simple: free radicals are bad, antioxidants must be good*” [50]. The huge amount of research carried in the past decades in the field provided a progressive change of the paradigm, namely, that “*too many oxidants are bad, but some may be good*” [44]. Undoubtedly, free radicals account for the harmful effects (oxidative attack of proteins, lipids, and DNA) only when rapidly generated in elevated concentrations (e.g., during the postischaemic reperfusion) whereas in low or moderate amounts they act as signaling molecules with a critical role in the regulation of several fundamental physiological and adaptive processes (including cardioprotection).

## 3. Mitochondria as Sources of Harmful ROS

### 3.1. Oxidative Stress: Old and New Definitions

Oxidative stress has been classically defined as the spatiotemporal, quantitative imbalance between increased ROS formation (prooxidant stress) and decreased ROS removal (antioxidant defense) that is responsible for cellular damage [51, 52]. It has to be mentioned that the term does not refer only to the overproduction of “true” free radicals (molecules containing one or more unpaired electrons), such as superoxide anion (the primary ROS) and hydroxyl radical, but also to the increased generation of highly reactive nonradical derivatives, mainly hydrogen peroxide, peroxynitrite, and singlet oxygen [53].

However, ten years ago Jones proposed a new definition of stress as being “*an imbalance between oxidants and antioxidants in favor of the oxidants, leading to a disruption of redox signaling and control and/or molecular damage*,” pointing to the crucial role of disrupting the ROS-mediated signal transduction [54]. Moreover, Jones challenged the free radical dependent-oxidative stress theory by postulating the radical-free “*redox hypothesis*” according to which oxidative stress occurs via the disruption of thiol pathways due to aberrant generation of nonradical oxidants in distinct subcellular compartments [55]. In this respect, the term has been recently redefined in that oxidative stress should be perceived rather as a subcellular deleterious event than as a global threat to the whole cell [53]. In any case, regardless of the theory, mitochondria are the organelles that lie at the heart of redox biology being at the same time the sources of harmful and beneficial ROS and the main targets for oxidation.

### 3.2. Mitochondrial Sources of Harmful ROS

Mitochondria consume about 98% of the inhaled oxygen in order to produce the energy required to sustain life [56]. The increased efficiency of the oxidative phosphorylation in eukaryote cells comes at a price of mitochondrial generation of ROS; thus, ROS production and lethal reperfusion injury appear to be both a sort of “necessary evil” [57, 58].

Mitochondria have been conventionally recognized as the major cellular source for ROS production. Indeed, several expert research groups have systematically studied along the years the mitochondrial origins of ROS; in this respect, the reader is referred to several comprehensive reviews of the topic [59–71]. Moreover, it has to be mentioned that an unbiased estimation of the contribution of mitochondrial sources to oxidative stress in living cells requires not only multiple ROS reporter molecules but also parallel assessment of parameters that may induce artifacts as well as testing conditions that could interfere with the mitochondrial generation of oxidants [72]. What is unequivocally established so far is that ROS can be generated* in vitro* as either an* accidental* or an* obligatory* by-product of mitochondrial metabolism [73]. The former case is best exemplified by a dysfunctional electron transport system (ETS) (due to toxic or stress-related inhibition, mutational damage, and elevation of mitochondrial membrane potential due to metabolic causes) whereas the latter is best exemplified by the increased activity/expression or assembly failure of enzymes with defined metabolic roles [51, 57, 73]. More recently, the group of Koopman suggested that local ROS and/or reactive nitrogen species (RNS) involved are short-term regulation of mitochondrial morphology (fusion and fission) and function via nontranscriptional pathways [74].

There is plethora of experimental evidence supporting the roles of complexes I and III of the ETC as major generators of superoxide, the primary ROS (reviewed in [26, 59–61, 64–66, 84] and summarized in Table 1). Interestingly, Forkink et al. have recently suggested that the increase in ROS levels is not surpassing the capacity of the antioxidant systems within the cells [153]. In this respect, they demonstrated that chronic inhibition of CI and CIII in HEK293 cells (i) stimulated oxidation of the ROS sensor hydroethidine, (ii) increased cytosolic (but not mitochondrial) H_2_O_2_ levels, and (iii) was not associated with oxidative stress or cell death [153].

In the past years, several studies have also demonstrated the role of complex II defect in ^∙^O_2_^−^ overproduction (Table 1). Among these, the groups of Quinlan et al. [98] and Siebels and Dröse [94] have studied ROS generation at complex II in artificial conditions, such as a low concentration of succinate and inhibition of respiratory chain downstream to CII [94, 98]. Finally, ROS generation by complex IV was demonstrated to be rather relevant in pathological conditions by Prabu et al. since hyperphosphorylation of complex IV on ischaemic hearts increases the electron leakage and, therefore, the ^∙^O_2_^−^ production [108].

In addition to the ETS, several other mitochondrial sites (see Table 1) can be also responsible for ROS production in a tissue-specific manner and dependent on the experimental conditions [64]. Moreover, one of the most pertinent observations has been recently formulated by Andreyev et al.; these authors acknowledged the fact that, in line with the observer effect postulated in quantum physics, directly assessing ROS production using the conventional systems is not possible without changing the process [57].

### 3.3. Mitochondrial ROS Generation in Ischaemic/Reperfused Heart

Physiological, low concentrations of mitochondrial ROS are considered to exert beneficial effects on cardiovascular function [97]. Accordingly, a tight redox control is responsible for cardiomyocyte differentiation and excitation-contraction coupling [154, 155]. On the contrary, ROS overproduction is responsible for the so-called phenomenon “ROS-induced ROS release” [70, 156] or the “kindling radicals” concept [157, 158], which postulates that (extra)mitochondrial ROS trigger mitochondrial ROS production, with a pathological impact on (1) cardiac cells via the cellular bioenergetic decline which leads to the impairment of excitation-contraction coupling, arrhythmias, cardiac hypertrophy, apoptosis, necrosis, and fibrosis [71]; (2) endothelial cells, with 2 major effects: (i) the inflammatory vascular reaction involved in the pathogenesis of atherosclerosis, hypertension, and diabetes [159]* via* the activation of Ca^2+^-activated potassium channel (KCa channel) coupled with intracellular signaling of PKG-1*α* activation in the smooth muscle cells [160, 161] and (ii) the coronary collateral growth inhibition [162]* via* the coronary dilation mediated by the activation of voltage-dependent potassium channels (Kv channels) and thiol redox-dependent signaling [163, 164].

**Figure 1:**
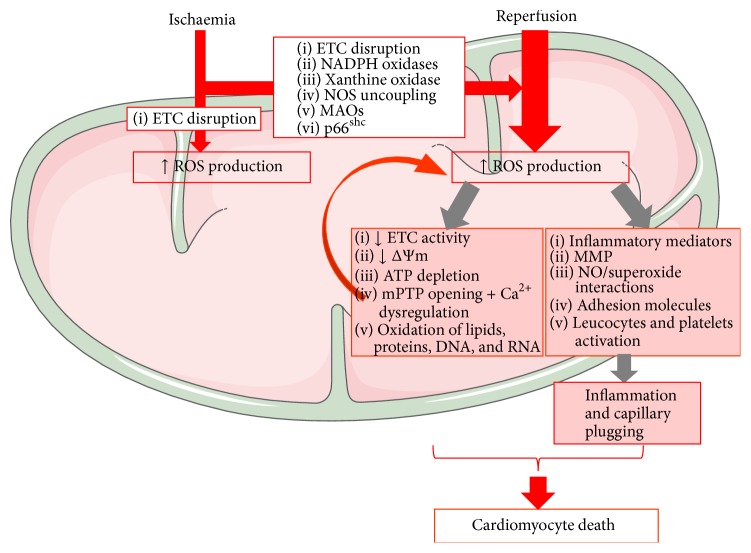
Mitochondrial ROS contribution to I/R injury. Cellular hypoxia secondary to ischaemia results in disruption of ETC activity in the IMM (inner mitochondrial membrane) with subsequent ROS production. Increased activity of MAOs, NADPH oxidase, and p66^shc^; conformational changes of xanthine oxidase; and/or NO synthase uncoupling further amplify ROS production upon reoxygenation. Increased mitochondrial ROS damages mtDNA and RNA with ETC impairment. Dysfunctional ETC will amplify ROS generation, leading to a vicious cycle of mitochondrial cumulative damage, decreased mitochondrial membrane potential (Δ*ψ*_*m*_) and respiration, mPTP opening with cellular swelling and Ca^2+^ dysregulation, and oxidation of lipids and proteins. Postischaemic ROS generation also stimulates an inflammatory response, with the release of chemical mediators and expression of adhesion molecules by endothelial cells and leukocytes. ROS-dependent activation of MMPs (matrix metalloproteinases) is also responsible for the functional impairment of several proteins and receptors. The inflammatory response and the activation of leucocytes and platelets trigger the narrowing of capillaries during reperfusion, accelerating the progression towards cardiomyocyte death. (Illustration realized thanks to Servier Medical Art.)

In the setting of I/R injury the contribution of mitochondria-derived ROS to oxidative stress is particularly true for the metabolically active organs, such as heart and brain [26]. Both hyperoxia (at reperfusion) and, also (albeit counterintuitively), hypoxia (during the ischaemic period) are able to trigger ROS production (Figure 1, [26, 68, 165]). During ischaemia, cardiomyocytes become hypoxic and the mitochondrial ETC complexes are highly reduced; the reaction of the electrons leaking from the respiratory complexes with residual oxygen will generate the superoxide anion. At reperfusion, hyperoxygenation will be associated with marked superoxide and superoxide-downstream ROS (mainly, hydrogen peroxide, peroxynitrite, and the hydroxyl radical) both due to electron leakage and due to a decrease in the detoxification capacity of mitochondria (Figure 1, [71]). An important consequence due to myocardial I/R is the change in the mitochondrial balance NO/^∙^O_2_^−^, with an increased NO production, subsequent excess of ONOO^−^ synthesis, and an increase of the related protein tyrosine nitration [166]. An enzyme with high susceptibility to oxidative stress is aconitase, whose activity is clearly impaired during myocardial I/R, followed by the increase of hydroxyl radicals release [167], an observation which could be suggestive for using the oxidative inactivation of mitochondrial aconitase activity as an additional marker of myocardial infarction [71].

The major contributor to ROS overproduction during I/R is related to the oxidative impairment of mitochondrial complex I along with a corresponding decrease in NADH-linked state 3 oxygen consumption and enhanced NADH-linked ROS production, respectively [168, 169], and reviewed in [23, 71]. During reperfusion, the NADH-ferricyanide reductase activity (the enzymatic activity of NADH dehydrogenase) is restored, which partially explains the ^∙^O_2_^−^ production during reperfusion, since NADH dehydrogenase is one of the major sources for ^∙^O_2_^−^ generation at complex I. Impaired complex I activity during reperfusion might be also responsible for ROS-induced damage of mitochondrial cardiolipin and respiratory supercomplexes that further increases the electron leakage at complex I and induces a vicious cycle of oxidative stress that ultimately leads to mitochondrial dysfunction [169, 170]. Lastly, an important protein tyrosine nitration of complex I in the postischaemic heart was demonstrated with a subsequent inactivation of complex I [166].

The involvement of complex II in ROS production in ischaemic hearts is unclear, despite the fact that diazoxide or atpenin A5 (specific complex II inhibitors) has been proven to exert cardioprotective effects by activating mitochondrial ATP-sensitive potassium (mK_ATP_) channels [71].

Complex III is also considered an important source for mitochondrial ROS production in ischaemic hearts [171], due to increased lipid peroxidation of cardiolipin required for complex III activity [172] and increased protein tyrosine nitration [166]. The electron leakage at complex III was associated with pharmacological preconditioning by diazoxide (the classic mitoK_ATP_ channel opener)* via* the inhibition of complex II with and transient generation of signaling ROS at complex III [173].

Lastly, complex IV-mediated ROS production can also be enhanced in ischaemic hearts* via* the activation of mitochondrial protein kinase A (PKA) which increased hyperphosphorylation of complex IV [108, 161]. Moreover, Spear et al. demonstrated that PKA-mediated depression of complex IV activity was reversed by blocking *β*1-adrenergic receptor activation during I/R, with a subsequent reduction of the myocardial injury [167].

Another recently investigated mitochondrial source for ROS generation is represented by monoamine oxidases (MAOs), two isoforms, MAO-A and MAO-B, located on the outer mitochondrial membrane. These FAD-containing dehydrogenases catalyze the electron transfer from the biogenic amines to O_2_ and constantly generate hydrogen peroxide (H_2_O_2_) as by-product. MAO-derived H_2_O_2_ is the primary signaling molecule when generated in minute amounts and becomes harmful when highly generated during conditions associated with oxidative stress [73]. Accordingly, in settings of postischaemic reperfusion or heart failure, the increased activity of MAO-A isoform significantly contributed to the aggravation of myocardial injury [174–177] and progression towards the maladaptive left ventricle hypertrophy and remodeling, respectively [125, 178]. These studies have unequivocally demonstrated the role of MAO-A in cardiac pathology; however, recent experimental data also reported the presence and contribution of MAO-B isoform to oxidative stress in the murine cardiovascular system [126, 128, 179]. We have recently demonstrated that both MAOs isoforms are expressed in atrial appendages harvested from patients with cardiovascular pathology (i.e., valvular disease and coronary heart disease), with the predominance of the MAO-B isoform (Duicu et al., in press). To date there is only one study in the literature showing that an increased activity of MAO-B is responsible for the induction of mitochondrial dysfunction and cardiac structural/functional alterations in mice with experimentally induced heart failure [126].

### 3.4. Antioxidant Strategies in Ischaemia/Reperfusion Injury

The mitochondrial antioxidant system is a network of high complexity (the reader is referred to several comprehensive reviews [51, 57, 143]) and comprises 3 major categories: (1) the first one includes superoxide dismutase 2 (MnSOD) and catalase, which exert their ROS neutralizing activity independent of the reducing equivalents; (2) the second one includes peroxiredoxins 3 and 5 (Prx3 and Prx5, located in the mitochondrial matrix), which depend on thioredoxin (Trx) and thioredoxin reductase (TRx2) for their regeneration; (3) the third one includes glutathione peroxidases 1 and 4 (GPX1 and GPX4) and glutaredoxins, which depend on GSH and glutathione reductase (GR) to regenerate GSH [57]. The last two categories of ROS scavengers depend on NADPH, which in turn is regenerated by 3 mitochondrial matrix enzymes: isocitrate dehydrogenase (NADP^+^-linked), malic enzyme, and transhydrogenase [60]. The individual contribution of these enzymes to mitochondrial NADPH regeneration is far from being elucidated [180].

The mechanisms that potentially could underlie the mitigation of ROS generation are listed in Table 2.

Therapeutic antioxidant approaches against the I/R myocardial injury have been disappointingly ineffective [181–184] or even harmful [53], most probably because the applied strategies were not able to distinguish between deleterious and beneficial ROS generation [29] and these differences between animal and human pathological models [185]. However, recent data have proven an enhanced therapeutic efficiency of novel synthetic antioxidants in ameliorating the I/R-linked oxidative stress with a subsequent cardioprotective effect. Such antioxidants include NO-based and vitamin E (MitoVit-E) molecules which are able to sequester antioxidants in mitochondria and Alda 1, a small molecule activator of aldehyde dehydrogenase-2, a mitochondrial enzyme that detoxifies aldehydes involved in myocardial I/R (reviewed in [29]). In bovine aortic endothelial cells exposed to oxidative stress, MitoVit-E significantly decreased ROS production and apoptosis [186], yet it was not neuroprotective in striatal medium-spiny neurons subjected to acute perinatal hypoxic–ischaemic brain injury [187]. An important disadvantage of MitoVit-E is that its scavenging activity is not regenerated [188]. At variance, mitoquinone (MitoQ) containing the antioxidant coenzyme Q (quinone) is regenerated by ETC after detoxifying ROS and was proven to inhibit mitochondrial oxidative stress in rodent models of I/R [189]. Another synthetic mitochondrial scavenger is the plastoquinone SkQ1, which used in a lower concentration than that of MitoQ was also able to reduce the infarct size and arrhythmias in rats subjected to I/R [190].

Of a particular promise for the inhibition of deleterious ROS induced by I/R injury might be the gene therapy approaches as demonstrated so far by two recent studies that used target upregulation of mitochondrial antioxidant enzymes like MnSOD or matrix peroxiredoxins [191] or overexpressed prosurvival molecules such as aldehyde dehydrogenase-2 microRNAs [192].

## 4. Mitochondria as Sources of Beneficial ROS

Until the 80s, I/R injury was considered a black or white phenomenon, the cardiomyocytes being provided with no more than two options—recovery or death. However, starting with the 80s, it became apparent that myocardial cells exposed to a variety of insults, including ischaemia, have an innate ability to mount several cardioprotective responses and an inherent program for survival. In the early days of myocardial I/R research, it was found that while the reintroduction of oxygen through reperfusion was essential for recovery, this also caused a burst of free radicals finally leading to myocardial injury [193]. Based on the concept that ROS only have deleterious effects, the administration of free radical scavengers was thought to be an appropriate solution in this situation. Several studies reported protective effects of this strategy, which unfortunately were surprisingly not supported by other independent laboratories [194]. Nowadays, it is largely accepted that some ROS represent intracellular mediators in physiological processes like vasodilation, cell growth, and angiogenesis and redox signaling is an important determinant of epigenetic and genetic regulation of cellular function. It has now become abundantly clear that, in cardioprotection against I/R injury, ROS present a delicate beneficial to deleterious switch [28] and this cross talk to and from mitochondria [157] might be favorable since the inhibition of a single source of ROS partially or even completely abrogated the oxidative stress [127].

The most powerful intrinsic mechanism of cardioprotection is represented by ischaemic preconditioning (IPC) which has been reported to cause adaptation to ischaemia in, virtually, all experimental settings from cell cultures to mammals. This strategy was first established by Murry et al. in 1986 [195] and describes the ability of brief periods of nonlethal ischaemia alternated with reperfusion to protect the heart from a subsequent prolonged lethal or “index” ischaemia. In 2003, the term postconditioning (PostC) was coined by the group of Vinten-Johansen to define a series of brief mechanical interruptions of reperfusion that were early applied within the first 3 minutes of reperfusion, elicited an anti-infarct protection comparable to the one induced by IPC [196]. The introduction of this appealing term as a novel strategy to limit lethal reperfusion injury, even if criticized by some authors who considered it as a form of modified reperfusion or compared it to “an old wine in a new bottle” [197], has the huge merit of resuscitating the concept that more myocardium can be salvaged by adding adjunct therapies to the early reperfusion. IPC and PostC require direct intervention on the heart, which may be challenging in some clinical situations. Remote ischaemic conditioning (RIC) was developed as a procedure performed by applying brief cycles of nonlethal I/R in a vascular territory remote from the heart. Although it is similarly cardioprotective to IPC and PostC, the fact that it is implemented at a distance from the organ of interest constitutes an evident advantage [198]. All these strategies represent endogenous self-defense mechanisms that are dependent on ROS generation. The identification of the most relevant sources of ROS and the threshold at which they lose their potentially protective effect and become damaging to cellular function and integrity still represents an unmet need in the field of cardioprotection [27].

### 4.1. Ischaemic Preconditioning

To date, no other strategy aimed at reducing I/R injury has proven itself to be more effective than IPC and thousands of paper tackled the mechanisms underlying its protective effect with numerous signaling molecules being identified as participating in the signal transduction sequence [199]. Among these, the generation of sublethal amounts of ROS during the short cycles of ischaemia and/or reperfusion has been consistently reported to be the trigger of IPC, possibly through the oxidation of protective cytosolic kinases [200]. The direct consequence of minute ROS generation prior to the prolonged ischaemia was the triggering of a “ROS-induced ROS decrease” response during the postischaemic reperfusion in every species tested. The observation that the deleterious burst of ROS upon reperfusion is reduced when IPC is applied has been demonstrated 20 years ago [201] and is still valid until today [202]. In addition, administration of exogenous ROS induces a protective effect similar to IPC [203], whereas antioxidants decrease or abolish cardioprotection [204, 205].

Mitochondria have emerged as the major source of ROS generation within the cardiac myocytes in the setting of preconditioning [206]. The PKC*ε* activated by IPC induces the stimulation of mitochondrial K_ATP_ channels causing a slight increase in H_2_O_2_ production which eventually leads to the inhibition of the mitochondrial permeability transition pore (mPTP) [207], seemingly the final effector of IPC [208]. Within this cascade of events, there might be a direct interaction between ROS and mPTP components or the sublethal oxidative stress can set in motion signaling pathways that decrease mitochondrial susceptibility to mPTP opening [27]. Also, a small level of ROS can be generated through a brief opening of the mPTP that may play an important role in cardioprotection [209]. In line with this observation, it has been reported that the inhibition of CyP-D results in abolition of ROS formation and of IPC-related cardioprotection, respectively [210].

In addition, it may be that all forms of cardiomyocyte stress lead to ROS signaling and that this could represent a mechanism for gaining ischaemic tolerance, as data suggests that hyperthermic preconditioning is reliant on ROS production [211].

An important source of mitochondrial H_2_O_2_ generation is the activation of MAO-A (as discussed in Section 3) during reperfusion with the occurrence of apoptosis in isolated cardiac myocytes; indeed, in lower concentrations, H_2_O_2_ was found to be partly responsible for the cardioprotective effect of IPC [212]. Supporting this claim, MAO inhibition in the settings of IPC has been reported to abolish cardioprotection (Di Lisa, unpublished observations, cited by [213]). However, in a recent study in isolated rat hearts subjected to a preconditioning protocol, we have demonstrated that bracketing the IPC episodes with MAO inhibitors did not interfere with the antinecrotic protection but potentiated the postischaemic functional recovery [214].

### 4.2. Ischaemic Postconditioning

Even though IPC is indeed the most efficient strategy, postconditioning (PostC) also proved to afford cardioprotection, although slightly less so than the former in terms of decreasing the infarct size. Penna et al. were the first to notice that ROS are needed to trigger PostC-related protection too [215]. In line with this observation, other studies have proven that PostC-mediated protection was abolished in the presence of ROS scavengers at the beginning of reperfusion [215, 216].

Cardioprotection is also possibly mediated by the prevention of mPTP opening by acidosis during the PostC cycles, while the intermittent bursts of oxygen throughout the brief I/R episodes allow mitochondria to produce just enough ROS in a moment when other enzymes, responsible for the generation of massive quantities of free radicals, are not yet reactivated. The consequent activation of the PKC pathway leads to the sensitization of adenosine receptors, signaling via the RISK pathway [199], and, finally, the prevention of mPTP formation even after the pH returned to normal [213]. Although all the mentioned evidence supports the idea that PostC-related cardioprotection is dependent on redox signaling, it is also apparent that the type, concentration, and the sources of ROS may be key factors in triggering protection at the time of reperfusion [213]. Contrary to the limited clinical applicability of IPC, PostC applied to humans in the cardiac catheterization laboratory has provided encouraging results by two clinical studies [217, 218], whereas others found no cardioprotective effects [219, 220]. Such discrepancies might be the result of different inclusion/exclusion criteria and the differences in the PostC chosen protocols. Thereby, the results of the DANAMI-3 trial (NCT01435408) designed to investigate postconditioning in STEMI patients are awaited this year with real interest [221].

### 4.3. Remote Ischaemic Conditioning

Remote ischaemic conditioning (RIC) was firstly described in 1993 by Przyklenk et al. [222] who noted that brief episodes of I/R applied in one region of the heart are protective for remote virgin myocardium in a separate myocardial territory. The mechanisms behind RIC are very complex and occur in three interrelated stages: (1) the I/R stimulus induces the synthesis of protective factors in the remote organ; (2) the protective signal is transmitted through a complex neurohumoral interaction to the target organ; (3) the events taking place in the target organ result in the protective effect.

Presumably, the signaling pathways activated in the remote and the target organ, respectively, are similar to those described in IPC and PostC [198]. Again, ROS production is part of the signaling cascade involved. Once the cardioprotective signal resulting from the ischaemic remote organ reaches the heart, it binds to G-protein cell surface coupled receptors which activate intracellular kinases like PKC-*ε* and other signaling molecules such as ROS and the mitochondrial K_ATP_ channel [223]. It has been demonstrated that IPC and remote ischaemic preconditioning (RIPC) both rely on free radicals to induce cardioprotection, as* N*-2-mercaptopropionyl glycine, an antioxidant, is capable of completely blocking the beneficial effect of RIC when ischaemia is induced by infrarenal occlusion of the rat aorta [224]. Also, in a model of RIPC obtained by occlusion of the mouse femoral artery, the ischaemia applied in the remote organ induced* S*-nitrosation of mitochondrial complex I in cardiomyocytes, which resulted in a reduction of ROS (i.e., H_2_O_2_) in the reperfused myocardium at risk [225].

As in the case of PostC, RIPC has been successfully translated to humans and recent pilot studies showed that it is able to improve clinical outcome and prognosis (excellently reviewed by [221]). In order to confirm these proof-of-concept studies, two multicentre trials of RIPC are currently ongoing, namely, NCT01857414 (CONDI II trial) and NCT02342522 (ERIC-PPCI trial).

Despite the successful results in animal models (reviewed in [30]) and in several pilot human studies (see above), the results of the cardioprotective trials targeting mitochondria have been rather disappointing (Table 3).

## 5. Mitochondria-Derived ROS and Endothelial Dysfunction

The mitochondrial content in the endothelial cells is rather poor as compared to other cells, for example, 2–6% of the rat cell volume versus 28% in hepatocytes or 32% in cardiomyocytes [226, 227]. At variance from cardiomyocytes, endothelial mitochondrial content and energy requirements are relatively reduced, glycolysis being the main source of ATP production [228]. Nowadays these organelles are considered major players in both cell signaling and vascular disease [188]. Moreover, mitochondrial cellular distribution represents a key factor for its function. In this view, the group of Gutterman demonstrated in endothelial cells isolated from human coronary arterioles that mitochondria are anchored to the cytoskeleton being thus responsible for ROS release in response to cell deformation by shear stress [229]. Another relevant study sustaining this theory demonstrated that pulmonary artery exposed to hypoxia induced a retrograde mitochondrial movement requiring microtubules and the microtubule motor protein dynein, changes that lead to a perinuclear clustering of mitochondria [230]; moreover, this mitochondrial redistribution was associated with ROS accumulation in the nucleus, which was further reduced by nocodazole which destabilized the microtubules and, thus, suppressed the perinuclear clustering of mitochondria [230]. In recent years, an increasing attention has been payed to the alterations of mitochondrial fusion and fission, due to their harmful consequences on cellular bioenergetics and endothelial dysfunction in the settings of cardiovascular disorders [74, 231, 232].

A wealth of clinical and experimental studies unequivocally demonstrated that endothelial dysfunction represents a central event in the pathogenesis of cardiovascular diseases (recently reviewed in [233]). Risk factors, such as aging, hypercholesterolemia, hyperglycemia, smoking, infections, and hypoxia, alter the mitochondrial membrane potential (Δ*ψ*_*m*_), with a subsequent contribution to excessive mitochondrial ROS production [233]. If the membrane is depolarized, complexes I and III show an increased activity in order to restore membrane potential, thus leading to ROS generation [226]. Metabolic disease states associated with high nutrient availability and low ATP demand are characterized by membrane hyperpolarization which also results in excessive ROS [227]. The consequential modifications of mitochondrial components affect the mtDNA, proteins, and lipids which in turn will stimulate ROS production, creating thus a vicious cycle that promotes vascular disease [234, 235]. Moreover, the mtDNA damage is responsible for the alteration of the ETS components expression, leading to an increased ROS production [236].

Apart from complexes I and III of the respiratory chain, another important source of mitochondria-derived ROS in endothelial cells is nicotinamide adenine dinucleotide phosphate (NADPH) oxidase 4 (NOX-4), which is the most highly expressed Nox family member in the endothelial layer of vasculature, being localized in many intracellular compartments, including mitochondria [237, 238]. Endothelial cells and basal ROS production, mainly H_2_O_2_ (rather than ^∙^O_2_^−^) as stated by [239], require NOX-4 and its homolog NOX-2 [238]. More recently, it was suggested that NOX-4 has rather a preventive function, since it protected the vasculature during ischaemic or inflammatory stress [240]. Thus, the contribution of NOX-4 to ROS signaling, angiogenesis, oxidative stress, endothelial dysfunction, and inflammation processes is far from being fully elucidated [240–244].

Another mitochondrial source of ROS is the growth factor adapter protein p66^shc^. In physiological conditions, p66^shc^ is included in a high-molecular-weight inhibitory protein complex located in the mitochondrial matrix or even in the cytoplasm. Circumstances associated with proapoptotic signals, such as hypoxia, activate p66^shc^ which migrates in the mitochondrial intermembrane space, where, through the oxidation of cytochrome c, it generates H_2_O_2_ [116]. Moreover, p66^shc^ can become active via phosphorylation by protein kinase C in conditions associated with hyperglycemia, contributing thus to diabetic endothelial dysfunction [117, 245]. p66^shc^ deletion in models of vascular injury has yielded beneficial effects [117, 246], supporting thus the statement about its implication in oxidative stress [227].

Mitochondrial ATP-sensitive potassium channel (mitoK_ATP_) represents a regulator of mitochondrial free oxygen radicals. Although it has not been the focus of interest in vascular dysfunction so far, its activation seems to protect cultured endothelial cells from ischaemic cell death and to maintain vasodilating capacity in Langendorff-perfused guinea-pig hearts suffering from I/R injury [247, 248]. Furthermore, glibenclamide, a nonselective K_ATP_ channel blocker, abolished the IPC-induced preservation of endothelium-dependent dilation in the human forearm, while the mitoK_ATP_ opener diazoxide mimicked the IPC protection [249, 250]. These potassium channels also demonstrated the ROS-induced ROS release theory, since ROS produced by another cellular structure acted by opening mitoK_ATP_, stimulating the generation of mitochondrial ROS [157, 251]. It might be, thus, reasonable to assume that the inhibition of these channels might be protective. As in the case of cardiomyocytes, the phenomenon of ROS-induced ROS release can equally contribute to the pathogenesis of endothelial dysfunction [252].

Lastly, H_2_O_2_ generated by MAO-A in vascular smooth muscle cells contributes to serotonin-induced vasoconstriction [253]. Although it was clearly demonstrated that endothelial cells express MAOs [254], the exact role of these enzymes in modulation of endothelial function has not been fully characterized. Recently, we have described the role of MAOs as mediators of endothelial dysfunction in two murine models of acute (induced with lipopolysaccharide, LPS) and chronic (induced with angiotensin II, AII) [127] oxidative stress or after the induction of experimental diabetes [128]. Both isoforms increased the expression of vascular MAOs with subsequent high H_2_O_2_ generation. This mechanism was deemed responsible for the induction of oxidative stress, altered level of cGMP with a central role in NO-mediated signaling, and a consecutive impairment of aortic rings relaxation [127]. It is important to note that all changes induced by MAOs activation were reversed by the MAO-A and MAO-B inhibitors [127], proving thus the contribution of mitochondria-derived ROS to endothelial dysfunction.

Reperfusion following ischaemia is associated with an increased endothelial generation of ROS and endothelin and a reduced availability of nitric oxide. This latter event promotes neutrophil adhesion to the vascular endothelium and platelet aggregation, which, coupled with the effect of endothelin, will eventually lead to vasoconstriction which is responsible for the no-reflow phenomenon; the adhesion of neutrophils will further enhance ROS release from the endothelium and neutrophils [255]. ROS production represents, thus, an important path in mitochondrial signaling [227], which explains the huge interest in the elucidation of their sources and regulatory mechanisms.

Obviously, the nature of mitochondrial ROS signaling in endothelial cells is still a matter of debate. It may be that the significance of mitochondrial ROS in endothelial cell signaling varies according to vascular bed and risk factor burden [256]. Accordingly, there is evidence that dilation of human cardiac arterioles depends on ETC-derived ROS [229] and that antioxidant therapy blunted the dilation of the healthy human brachial artery [257]. On the other hand, in coronary arteries of atherosclerotic patients, H_2_O_2_ scavengers improved endothelium-dependent dilation [258], an effect also noticed in diabetic freshly isolated arterioles treated with mitochondria-targeted antioxidants [259].

In conclusion, targeting vascular ROS definitely represents an important research direction in order to alleviate endothelial dysfunction [260].

## 6. Conclusion

Despite the unequivocal beneficial effects of reperfusion in ceasing the progression of irreversible damage, it is largely accepted nowadays that (i) reperfusion is a double-edged sword as it is able to induce* per se* the myocardial lethal reperfusion injury which paradoxically alleviates the beneficial effects of revascularization and (ii) there is an unmet need and a strong interest in developing clinically effective cardioprotective interventions, which are able to further reduce infarct size in association with revascularization procedures. Mitochondrial dysfunction and the resulting oxidative stress are central in the pathogenesis of I/R injury and the drugs that can antagonize cardiomyocyte death by modulating mitochondrial function have started to be systematically investigated in clinical setting. Indeed, novel antioxidant compounds selectively targeting mitochondria appear to be an effective strategy to protect the heart against the deleterious effects of both ischaemic and reperfusion injury, two sides of the same coin. However, since the signaling mechanisms mediating I/R-induced mitochondrial dysfunction are diverse, a combination of pharmacological compounds or coadministration of drugs acting simultaneously on distinct targets should be envisaged.

## Figures and Tables

**Table 1:** Mitochondrial sources of ROS generation.

ROS sources	Experimental model: references
Inner membrane	
CI (NADH dehydrogenase): *inner side*	(i) Bovine hearts: [75–83] (ii) Rat heart: [80, 84–86] (iii) Rat brain: [60, 86, 87] (iv) Rat lung: [88] (v) Rat liver: [84] (vi) Rat skeletal muscle: [84, 89–91] (vii) Cell cultures: [79] (viii) Human brain: [87]
CII (succinate dehydrogenase): *inner side*	(i) Rat heart: [92, 93] (ii) Bovine heart: [94–96] (iii) Rat brain: [97] (iv) Rat skeletal muscle: [98] (v) Yeast: [99] (vi) *E. coli*: [100]
CIII (ubiquinol-cytochrome c reductase): *inner and outer side*	(i) Bovine heart: [95, 96, 101, 102] (ii) Rat heart: [84, 103, 104] (iii) Rat liver: [84, 105] (iv) Rat brain: [104] (v) Rat skeletal muscle: [84] (vi) Mouse skeletal muscle: [106] (vii) *R. capsulatus* strains: [107]
Hyperphosphorylation of CIV (cytochrome *c* oxidase)	(i) Rabbit hearts and mouse monocyte macrophages: [108]
Glycerophosphate dehydrogenase (a.k.a. glycerol-3-phosphate dehydrogenase, a.k.a. mGPDH): *outer side*	(i) Mouse heart, brain, and kidney: [109] (ii) Hamster brown adipose tissue: [110] (iii) *Drosophila*: [111]
Dihydroorotate dehydrogenase (DHO): *outer side*	(i) Rat brain & liver: [112] (ii) Rat skeletal muscle: [113] (iii) Rat tissues (skeletal muscle, liver, GI tract, etc.): [114] (iv) Cell lines: [115] (v) Human skin and kidney: [114]

Intermembrane space	
p66^Shc^ (growth factor adaptor Shc)	(i) Mouse liver: [116] (ii) Mouse aorta: [117]

Matrix	
Aconitase (mitochondrial- (m-) aconitase)	(i) Bovine heart: [118]
Alpha-ketoglutarate dehydrogenase complex (KGDHC, a.k.a. 2-oxoglutarate dehydrogenase)	(i) Bovine heart: [119] (ii) Mouse brain: [120]

Outer membrane	
Cytochrome *b*5 reductase	(i) Human brain tissue: [121]
Monoamine oxidases (MAO-A and MAO-B)	(i) Rat brain: [122] (ii) Rat hearts: [123] (iii) Mouse liver, kidney, and heart: [124–126] (iv) Mouse aorta: [127] (v) Rat aorta: [128] (vi) Cell line: [129] (vii) Human atrial samples: [130]

**Table 2:** Potential mechanisms responsible for the decrease in ROS generation.

Site of action	Mechanism
(1) UCP2 or UCP3 overexpression [131–133]	Reduced mitochondrial ROS production *via* mitochondrial uncoupling with subsequent Δ*ψ* depolarization

(2) Brief transient mPTP opening [134]	Reduced ROS production and/or release into the cytosol *via* a reversible Δ*ψ* depolarization *Observation: a prolonged mPTP opening triggers apoptosis and cell death* [135, 136]

(3) Recruitment of hexokinase (HK) at the mitochondrial outer membrane [137]	Increased coupled respiration with subsequent reduced electron leak and ROS production

(4) Glutathionylation of CII and CV [92, 138, 139]	Decreased activity of CII and CV

(5) Glutathionylation of the 51-kDa (NDUFV1) and 75-kDa (NDUFS1) CI subunits [79, 81, 140, 141]	Decreased activity of CI *Observation: however, CI inactivation is not necessarily linked to reduced ROS production since Taylor and collaborators demonstrated that glutathionylation of CI was associated to increased superoxide production* [142]

(6) Reduction of electrons input [143, 144]	Lowered cellular glucose uptake and stimulation of pyruvate conversion to lactate with secretion of the latter into the extracellular environment

(7) Mild uncoupling [145, 146] and inhibition of succinate dehydrogenase [147] *via *the action of potassium channel openers	Inhibition of CI with subsequent reduction of H_2_O_2_ release into the cytosol

**Table 3:** Cardioprotective strategies targeting mitochondria in clinical trials.

Trial	Strategy	Results
NCT01502774 (CIRCUS trial)	A bolus injection of CsA administered at the onset of myocardial reperfusion in patients with anterior ST-segment-elevation MI (STEMI)	Worsened heart failure during the initial hospitalization, rehospitalization for heart failure, and adverse left ventricular remodeling at 1 year in 59.0% of the 395 patients randomized to cyclosporine and 58.1% of the 396 individuals randomized to placebo [148]

NCT01374321 (MITOCARE trial)	I.v. bolus administration of TRO40303 (an inhibitor of mPTP opening) in STEMI patients undergoing primary PCI (percutaneous coronary intervention)	TRO40303 did not show any protective effects as compared to placebo in preventing reperfusion injury in STEMI patients treated with primary PCI [149]

NCT01572909 (EMBRACE STEMI trial)	MTP-131 (a cell-permeable peptide that preserves the integrity of cardiolipin, enhances mitochondrial energetics, and improves myocyte survival during reperfusion in animal models) administration for 1 h among first-time anterior STEMI subjects undergoing primary PCI for a proximal or mid left anterior descending (LAD) artery occlusion	Administration of MTP-131 was not associated with a significant reduction in infarct size or clinical outcomes [150]

NCT01584453 (NITRITE-AMI trial)	Intracoronary injection of nitrite during primary PCI in STEI patients	The phase II showed that intracoronary nitrite infusion did not change the infarct size. Yet, in a subgroup of patients with TIMI flow ≤1, nitrite reduced infarct size and MACE and improved myocardial salvage index indicating a follow-up with the phase III of the clinical trial [151]

NCT01388504 (NIAMI trial)	Intravenous sodium nitrite administration immediately prior to PCI in patients with acute STEMI	Myocardial infarct size did not differ between nitrite and placebo groups. There were no significant differences in plasma troponin I and CK area under the curve, left ventricular volumes, and ejection fraction measured at 6–8 days and at 6 months and final infarct size measured at 6 months [152]
